# Oxidative DNA Damage in Kidneys and Heart of Hypertensive Mice Is Prevented by Blocking Angiotensin II and Aldosterone Receptors

**DOI:** 10.1371/journal.pone.0115715

**Published:** 2014-12-31

**Authors:** Susanne Brand, Kerstin Amann, Philipp Mandel, Anna Zimnol, Nicole Schupp

**Affiliations:** 1 Institute of Pharmacology and Toxicology, University of Würzburg, Würzburg, Germany; 2 Institute of Toxicology, University of Düsseldorf, Düsseldorf, Germany; 3 Department of Pathology, University of Erlangen-Nürnberg, Erlangen, Germany; Max-Delbrück Center for Molecular Medicine (MDC), Germany

## Abstract

**Introduction:**

Recently, we could show that angiotensin II, the reactive peptide of the blood pressure-regulating renin-angiotensin-aldosterone-system, causes the formation of reactive oxygen species and DNA damage in kidneys and hearts of hypertensive mice. To further investigate on the one hand the mechanism of DNA damage caused by angiotensin II, and on the other hand possible intervention strategies against end-organ damage, the effects of substances interfering with the renin-angiotensin-aldosterone-system on angiotensin II-induced genomic damage were studied.

**Methods:**

In C57BL/6-mice, hypertension was induced by infusion of 600 ng/kg • min angiotensin II. The animals were additionally treated with the angiotensin II type 1 receptor blocker candesartan, the mineralocorticoid receptor blocker eplerenone and the antioxidant tempol. DNA damage and the activation of transcription factors were studied by immunohistochemistry and protein expression analysis.

**Results:**

Administration of angiotensin II led to a significant increase of blood pressure, decreased only by candesartan. In kidneys and hearts of angiotensin II-treated animals, significant oxidative stress could be detected (1.5-fold over control). The redox-sensitive transcription factors Nrf2 and NF-κB were activated in the kidney by angiotensin II-treatment (4- and 3-fold over control, respectively) and reduced by all interventions. In kidneys and hearts an increase of DNA damage (3- and 2-fold over control, respectively) and of DNA repair (3-fold over control) was found. These effects were ameliorated by all interventions in both organs. Consistently, candesartan and tempol were more effective than eplerenone.

**Conclusion:**

Angiotensin II-induced DNA damage is caused by angiotensin II type 1 receptor-mediated formation of oxidative stress *in vivo*. The angiotensin II-mediated physiological increase of aldosterone adds to the DNA-damaging effects. Blocking angiotensin II and mineralocorticoid receptors therefore has beneficial effects on end-organ damage independent of blood pressure normalization.

## Introduction

The epidemiological evidence of a relationship between hypertension and the risk to develop cancer is increasing. An early meta-analysis showing increased cancer mortality among hypertensive patients, as well as an almost doubled risk to develop renal cell cancer [1], was confirmed in the meantime [2], [3]. In addition, higher risks for bladder cancer, prostate cancer and breast cancer in hypertensive patients were identified [4]–[6]. First evidence now points to an association between the risk for renal cell cancer and the renin-angiotensin-aldosterone-system (RAAS) [7]. This hormone system regulates blood pressure via vasoconstriction of peripheral vessels and modification of sodium retention in the kidney. The effectors of the RAAS are angiotensin II (AngII) and aldosterone.

We could show *in vitro*, that both hormones, AngII and aldosterone, have a genotoxic potential [8], [9]. They cause DNA single and double strand breaks, abasic sites and increase the abundance of the DNA base modification 7,8-dihydro-8-oxo-guanine (8-oxodG) [10], [11]. Similarly, DNA lesions could be found in kidneys of animals with experimental hypertension, either caused by AngII- or aldosterone-infusion [12], [13]. As the underlying mechanism, we found *in vitro* the activation of reactive oxygen species (ROS)-generating enzymes like NADPH oxidase, via either the AngII type 1-receptor (AT1R) or the mineralocorticoid receptor (MR) [11], [14]. By administration of AT1R- and MR-blockers, as well as a ROS-scavenger, the mechanism of hypertension-induced genomic damage was studied here in mice with AngII-induced hypertension. By application of these three different agents known to interfere with the RAAS and blood pressure regulation, additionally pathways are explored, which might be targets for a reduction of the end-organ damage inflicted by AngII via the oxidative damaging of DNA.

## Methods

### Animal treatment

Male C57BL/6-mice (Janvier, Le Genest Saint Isle, France) at the age of 17 weeks were randomly distributed to five different groups with seven animals each (except the angiotensin II-treated group: n = 8), and were equipped under general anesthesia (ketamine 90 mg/kg and xylazine 6 mg/kg i.m.; medistar, Ascheberg, Germany) with osmotic mini pumps (Alzet, Model 1004; Durect Corporation, Cupertina, USA) delivering AngII (Calbiochem, Darmstadt, Germany) in a concentration of 600 ng/kg x min for 27 days. Control animals received the solvent PBS. In addition to the treatment with AngII, three groups were treated with: candesartan (8–10 mg/kg x d), an AT1R antagonist, tempol (4-hydroxy-2,2,6,6-tetramethylpiperidine-1-oxyl, 1 mmol/l), a radical scavenger in the drinking water, and eplerenone (100 mg/kg x d), an MR blocker, administered in rodent chow (ssniff, Soest, Germany). Blood pressure was measured via the non-invasive tail cuff method (Visitech Systems, Apex, NC, USA). At day 0 and day 27, mice were placed into metabolic cages and urine was collected during 20 hours for assessment of the renal function of the animals.

During the treatment time, 3 animals were lost due to infections, two of the eplerenone group and one of the tempol group. After 27 days of treatment the left ventricle was cannulated and the organs of the animals were perfused with Deltadex 40 (Deltaselect, Dreieich, Germany) containing 1% procainhydrochloride (Steigerwald, Darmstadt, Germany), followed by ice-cold 0.9% NaCl solution (Fresenius, Bad Homburg, Germany) in deep ketamine/xylazine anesthesia (ketamine 120 mg/kg and xylazine 8 mg/kg i.m.). Kidneys and heart were removed and parts were either embedded in paraffin or shock-frozen in liquid nitrogen.

All animal experiments were performed in accordance with the European Community guidelines for the use of experimental animals and with the German law for the protection of animals. The investigation conforms to the “Guide for the Care and Use of Laboratory Animals” published by the US National Institutes of Health (NIH Publication No. 85–23, revised 1996). The protocol was approved by the Regierung von Unterfranken, Würzburg (Permit number 55.2-2531.01-65/09).

### Immunohistochemistry

Immunohistochemistry was performed as described recently [12], with the following primary and secondary antibodies: anti-NF-κB p65 (sc-109, Santa Cruz Biotechnology, Santa Cruz, CA, USA), anti-PADPR (ab14460, abcam, Cambridge, UK) anti-Nrf2 (sc-7200, Santa Cruz Biotechnology), donkey anti-rabbit IgG-B and goat anti-chicken IgY-B (sc-2089 and sc-2430, Santa Cruz Biotechnology). For signal amplification of PADPR und Nrf2, the Tyramide Signal Amplification Biotin System (NEL700A001kit, Perkin Elmer, Whatman, USA) was used according to the manufacturer's instructions. Antibody binding was detected using a diaminobenzidine kit (SK-4100, Vector Lab, Burlingame, CA, USA). Sections were counterstained with hematoxylin. Pictures were taken with an Eclipse 55i microscope (Nikon, Düsseldorf, Germany) at 200-fold magnification. The ratio of positive cells/negative cells was assessed by the cell image analysis software CellProfiler [15] within seven visual fields.

### Immunofluorescence

For γ-H2AX-staining of the kidney, paraffin sections (2 µm) were deparaffinized using Roti-Histol (Roth, Karlsruhe, Germany) and ethanol. Antigen retrieval was performed with 0.01 mol/l citrate buffer, pH 6.0 at 95°C for 15 min. Incubation with the primary antibody (anti-γ-H2AX, # 9718, Cell Signaling, Herts, UK) was performed overnight at 4°C, followed by the addition of the secondary antibody: Cy3-conjugated goat anti-rabbit IgG (JacksonImmuno-Research Laboratories). Sections were counterstained with bisbenzimide. Pictures were taken with an Eclipse 55i microscope (Nikon, Düsseldorf, Germany) at 200-fold magnification. For γ-H2AX-staining of the heart, paraffin sections (2 µm) were stained as described [12]. For quantification of γ-H2AX-positive cells, the kidney and heart area was measured by using the point-counting method with a 121-point eyepiece (Leica, Wetzlar, Germany) at a 100-fold magnification. The number of γ-H2AX-positive cells was referred to the kidney/heart area.

### Western blot

Kidney tissue or heart tissue isolated from cryo-samples was manually pestled in liquid nitrogen and lysed in RIPA buffer (50 mM Tris, 150 mM NaCl, 1 mM EDTA, 0.025% Natriumdesoxycholat, 1% Nonidet, 1 mM NaF) supplemented with a protease inhibitor cocktail (Sigma, Taufkirchen, Germany) and the Halt phosphatase inhibitor cocktail (Thermo Scientific, Rockford, USA). Extracts were prepared by centrifugation at 8000 x g for 5 min. 50 µg of protein was loaded on SDS gels and separated proteins were transferred to polyvinylidene difluoride membranes (Roti-PVDF, Roth, Karlsruhe, Germany). Membranes were incubated with primary antibodies overnight: heme oxygenase-1 (HO-1), # 2322-1; NADPH oxidase isoform 4 (Nox4), # 3187-1 (Epitomics, Burlingame, USA), Xanthine oxidase (XO), # ab109235 (abcam, Cambridge, UK), superoxide dismutase (SOD) # GTX100554 (GeneTex, Irvine, USA), GAPDH, # 2118 (Cell Signaling, Herts, UK), followed by 1 hour incubation with horseradish peroxidase-conjugated secondary antibodies. Detection of bound antibodies was performed with an ECL Supersignal West Dura Detection kit (Thermo Scientific, Rockford, USA) according to the manufacturer's instructions. Chemiluminescent signals were recorded using Hyperfilm (Amersham, Buckinghamshire, UK), or using the Fusion FX7 imaging system (Peqlab, Erlangen, Germany).

### mRNA isolation and quantitative RT-PCR

Total RNA was isolated from kidneys by using the RNeasy Mini Kit (Qiagen, Hilden, Germany), according to the manufacturer's instructions. mRNA was reverse transcribed to cDNA using the Omniscript Reverse Transcription Kit (Qiagen, Hilden, Germany). Quantitative PCR was performed using the SensiMix SYBR Hi-ROX Kit (Bioline GmbH, Luckenwalde, Germany) on a StepOne Real-time PCR System (Applied Biosystems, Foster City, USA). For primer design, Primer Express software (Applied Biosystems) was used. Primer sequences used for gene expression analysis are listed in Table 1. Relative expression levels of target genes were normalized to (PPIA) and calculated by the use of the comparative CT method.

**Table 1:** Primer sequences used for real-time PCR.

Primer	Forward	Reverse
PPIA	GCGTCTCCTTCGAGCTGTTT	AAGTCACCACCCTGGCACAT
Nox1	AGGTCGTGATTACCAAGGTTGTC	AAGCCTCGCTTCCTCATCTG
Nox2	GGTTCCAGTGCGTGTTGCT	GCGGTGTGCAGTGCTATCAT
Nox4	CCCAAGTTCCAAGCTCATTTCC	TGGTGACAGGTTTGTTGCTCCT

NADPH oxidase isoform 1,

Nox2  =  NADPH oxidase isoform 2, Nox4  =  NADPH oxidase isoform 4.

### Parameters of renal function

Renal function was assessed by determination of creatinine clearance. Therefore creatinine in serum and urine was measured according to standard procedures using a Cobas analyzer (Roche, Mannheim, Germany) in the Institute of Clinical Biochemistry and Pathobiochemistry of the University of Würzburg. For quantification of albumin excretion, the Mouse Albumin ELISA Kit (EMA 3201-1, Assay Pro, St. Charles, USA) was used according to the manufacturer's protocol. The absorbance was read at 450 nm with an ELISA reader (Spectra MAX 340, Molecular Devices, Ismaning, Germany) and a standard curve was generated on each plate.

### Histopathology

For histopathological investigation of the kidney, 2 µm paraffin sections were cut and stained with hematoxylin and eosin (HE), periodic acid-Schiff stain (PAS) and Sirius red stain. In the kidneys the glomerular sclerosis index (GSI), the mesangiolysis index (MSI), the tubulointerstitial sclerosis index (TSI) and the vascular sclerosis index (VSI) were determined as described [16].

### Oxidative stress measurement

ROS production on cryosections was detected with dihydroethidium (DHE) and quantified as described earlier [13].

### Quantification of aldosterone

Urinary aldosterone excretion was measured using the Aldosterone ELISA Kit (BT E-5200, BioTrend, Köln, Germany), according to the protocol provided by the manufacturer. The absorbance was read at a wavelength of 450 nm with an ELISA reader (Spectra MAX 340, Molecular Devices, Ismaning, Germany), and a standard curve was generated on each plate.

### Quantification of 8-oxodG and 8-oxoGuo in urine

Mouse urine was thawed at room temperature, slightly mixed and diluted 1∶1 with the internal standard ^15^N_5_-dG (Silantes, Munich, Germany), dissolved in 50 mM lithium acetate buffer pH 6.4, yielding an end concentration of 250 nM ^15^N_5_-dG in each sample. Precipitates were dissolved by shaking for 10 min in a Thermo mixer at 37°C and 1000 rpm, followed by 10 min centrifugation at 10,000 rpm and 20°C. This procedure releases 8-oxodG and 7,8-dihydro-8-oxo-guanosine (8-oxoGuo) from precipitates [17], [18].

Measurement was conducted in an Agilent 1100 series HPLC equipped with LC Quaternary pump, solvent cabinet, degasser and autosampler (Agilent Technologies, Böblingen, Germany). For detection an Applied Biosystems Q-TRAP 2000 with turboionspray, controlled by Analyst Software 1.4.42, was used. A fully automated software controlled valco valve (VICI, Switzerland) diverted the early eluting components to waste, thereby reducing contamination of the ion source.

Separation was accomplished with a Phenomenex Luna 3 µ Phenyl-hexyl column (150×4.6 mm, 3 µ), protected with a Phenyl security guard column (4×3 mm), both obtained from Phenomenex (Aschaffenburg, Germany). The mobile phase contained eluent A (10 mM ammonium formate, pH 3.75) and eluent B (10 mM ammonium formate, pH 3.75 and 50% acetonitrile). The separation took place with a gradient elution, a flow rate of 200 µl/min and an injection volume of 25 µl. For the first 10 minutes 100% eluent A was used to wash out contaminations, after that the gradient was run over 30 min up to 70% eluent B. For 3 min 100% eluent B was injected and the column was re-equilibrated for 17 min with 100% eluent A. Conditions of detection with the API Q-TRAP 2000 (AB SCIEX, USA) during the first 43 min: ion spray voltage 4000 V, temperature 400°C and vacuum of 4.3*10e^−5^ Torr. As curtain, nebulizer and collision gas, nitrogen was used. Electrospray ionisation was performed with multiple reaction measurement in the positive mode; in Table 2 the different potentials for the analytes are listed. For the multiple reaction measurement of all analytes the [M+H]^+^ was selected by the first filter (Q1). After collision activation ions corresponding to the protonated nucleobase [BH_2_]^+^ were selected by the last mass filter (Q3) for nucleosides and deoxynucleosides. The following transitions were used for quantification: 8-oxoGuo (*m/z* 299.9-168), 8-oxodG (*m/z* 283.9-168) and ^15^N_5_-dG (*m/z* 273.2-157.1). Individual tuning files were produced to find the maximum sensitivity for each compound. For quantification a reference series for 8-oxodG und 8-oxoGuo in deionized water was prepared. The standards were diluted 1∶1 with ^15^N_5_-dG dissolved in 50 mM lithium acetate buffer (pH 6.4). The standards were measured three times simultaneously to the sample measurement.

**Table 2:** Multiple reaction monitoring parameters for the analysis of oxidized bases and internal standard and optimized conditions of the mass spectrometry measurement with the Q-Trap 2000.

Analyte	Transition [m/z]	Declustering potential [V]	Entrance potential [V]	Collision energy [V]	Collision cell exit potential [V]
	[M+H]^+^ (Q1) → [BH2]^+^ (Q3)				
8-oxoGuo	299.9 → 168	41	9.5	21	4
8-oxodG	283.9 → 168	31	8	19	4
^15^N_5_-dG	273.2 → 157.1	16	9	17	4

### Statistics

The data from 5–8 animals are shown as mean ± standard error mean (SEM). The data were tested for normality with the Kolmogorov-Smirnov test using SPSS 21 (IBM, Somer, USA). Normal distributed data was tested with analysis of variance (ANOVA) and subsequent post-hoc two-sided comparisons to the control or to the AngII group by Bonferroni were performed. Non-normal distributed data were tested with the Kruskal-Wallis test for significance among multiple groups and the Mann-Whitney-U test was used to determine significance between two groups. A p value ≤0.05 was considered significant. Raw data used to generate tables and figures are fully available in S1 Table.

## Results

### Blood pressure changes and clinical characteristics

**Figure 1:**
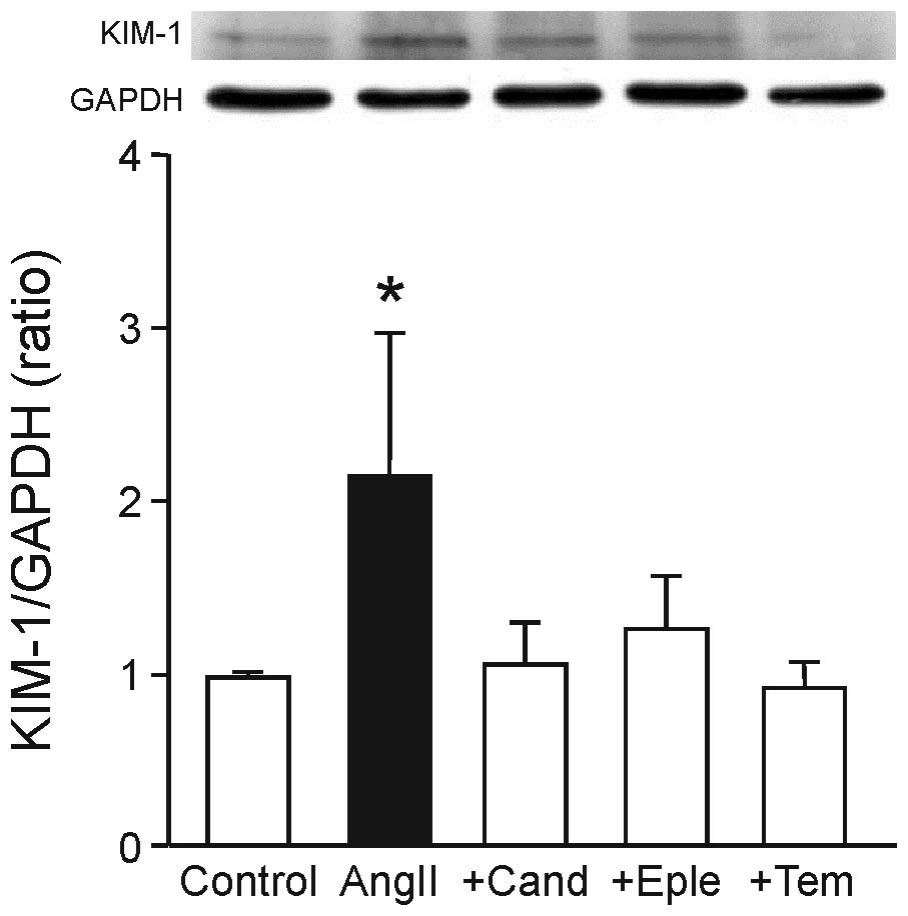
Induction of a marker of renal damage. Western blot-analysis of the amount of the kidney injury marker KIM-1 (kidney injury molecule) in kidneys of control animals and animals treated with angiotensin II (AngII) with or without co-treatment with candesartan (+Cand), eplerenone (+Eple), or tempol (+Tem). Shown is a representative blot and the quantification of band densities of proteins of all animals.* p≤0.05 vs. Control.

The chosen AngII dose of 600 ng/kg x min resulted in a significantly increased blood pressure in the AngII group (Table 3). While candesartan lowered the blood pressure to control values, eplerenone and tempol had no lowering effect. The body weight of mice treated with AngII with or without interventions was not significantly changed compared to the control group (Table 3). Kidney and heart weight was not affected, except in the candesartan group, which had a significantly lower heart weight compared to the AngII-treated group. Animals treated with AngII displayed an impairment of their kidney function, characterized by increased serum creatinine levels, a decreased creatinine clearance and significantly increased albumin levels, which were only ameliorated by candesartan (Table 3). Further, the kidney injury marker KIM-1 was significantly higher in kidneys of animals treated with AngII (Fig. 1). Aldosterone levels were increased in all groups compared to the control, except in the candesartan group, reflecting activation of the RAAS (Table 3).

**Table 3:** Clinical parameters, parameters of kidney function as well as aldosterone levels of mice after 27 days of angiotensin II-infusion.

	Control	Angiotensin II	Candesartan	Eplerenone	Tempol
Parameter	(n = 7)	(n = 8)	(n = 7)	(n = 5)	(n = 6)
SBP [mm Hg]	111±2	156±3***	104±5°°°	162±8**	165±6**
DBP [mm Hg]	90±3	115±5*	87±7°	132±12**	142±7***
Body weight	31±0.7	28±0.6	32±0.6	29±0.8	29±0.5
Left kidney weight [mg]	186±5	171±9	187±9	166±7	175±7
Heart weight [mg]	214±18	239±8	177±10°	233±22	239±12
Diuresis [ml/20 h]	1.7±0.3	3.9±0.2***	1.3±0.1°°°	2.8±0.4*,°	2.3±0.3°°
Serum creatinine [mg/ml]	0.13±0.02	0.17±0.02	0.11±0.01°	0.16±0.02	0.12±0.02°
Creatinine clearance [ml/h]	20±4	13±2	16±2	11±3*	15±3
Albumin/creatinine [µg/mg]	51±3	694±98**	52±4°°	509±63**	551±120***
Aldosterone/creatinine [ng/mg]	10±1	42±7***	11±1°°	58±4***	36±6*

* p≤0.05, ** p<0.01, *** p<0.001 vs. Control, ° p≤0.05, °° p<0.01, °°° p<0.001 vs. Ang II treatment.

### Histopathological changes of the kidney

**Figure 2:**
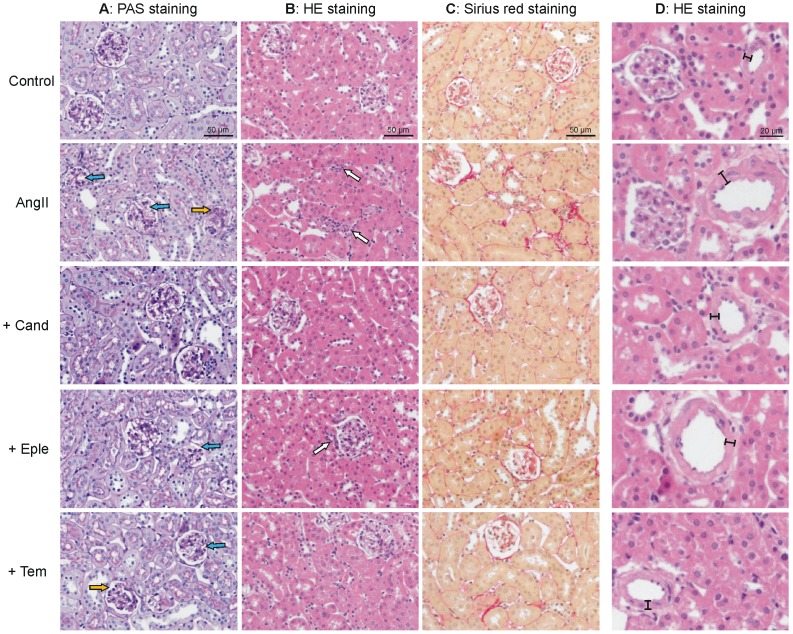
Histopathological changes of kidneys of control animals and animals treated with angiotensin II (AngII) with or without co-treatment with candesartan (+Cand), eplerenone (+Eple), or tempol (+Tem). A: Representative pictures of PAS-stained tissue, visualizing glomerular damage. B: Representative pictures of HE-stained tissue, visualizing regions of inflammation. C: Representative pictures of Sirius red-stained tissue visualizing regions of fibrosis (red). D: Representative pictures of HE-stained tissue, focussing on changes of the vasculature. Blue filled arrows: examples of mesangiolysis, orange filled arrows: examples of glomerulosclerosis, white filled arrows: examples of infiltrated leukocytes as a marker of inflammation, I–I: device illustrating the thickness of the vessel walls.

Glomeruli of mice treated with AngII showed structural changes in the form of increased mesangial cell size, matrix deposition and mesangiolysis with capillary dilatation (Fig. 2). Candesartan prevented the glomerular damage almost completely, while eplerenone and tempol were not as effective (Table 4). Pathological changes in the tubular system were characterized by tubular atrophy, occasional tubular dilatation and interstitial fibrosis, while centers of inflammation were almost not present in the kidney cortex. All interventions did significantly reduce the tubular damage. Treatment with AngII further led to vascular injury, expressed as thickening of vessel walls, which was ameliorated by all interventions (Table 4).

**Table 4:** Histopathological parameters.

	Control	Angiotensin II	Candesartan	Eplerenone	Tempol
Score	(n = 7)	(n = 8)	(n = 7)	(n = 5)	(n = 6)
GSI	1.0±0.1	2.5±0.2***	1.0±0.2°°°	2.0±0.3*	1.6±0.1°
MSI	1.0±0.1	3.0±0.3***	1.8±0.2°	2.1±0.1	2.4±0.4**
TSI	1.0±0.2	2.6±0.3**	1.2±0.1°°°	1.2±0.2°°	1.5±0.1°°
VSI	1.0±0.1	3.3±0.1***	1.5±0.2°°°	2.0±0.1***,°°°	1.8±0.1**,°°°

GSI: glomerular sclerosis index, MSI: mesangiolysis index, TSI: tubulointerstitial sclerosis index, VSI: vascular sclerosis index, each normalized to the Control values.

* p≤0.05, ** p<0.01, *** p<0.001 vs. Control, ° p≤0.05, °° p<0.01, °°° p<0.001 vs. Ang II treatment.

### Oxidative stress induced by angiotensin II in kidney and heart

**Figure 3:**
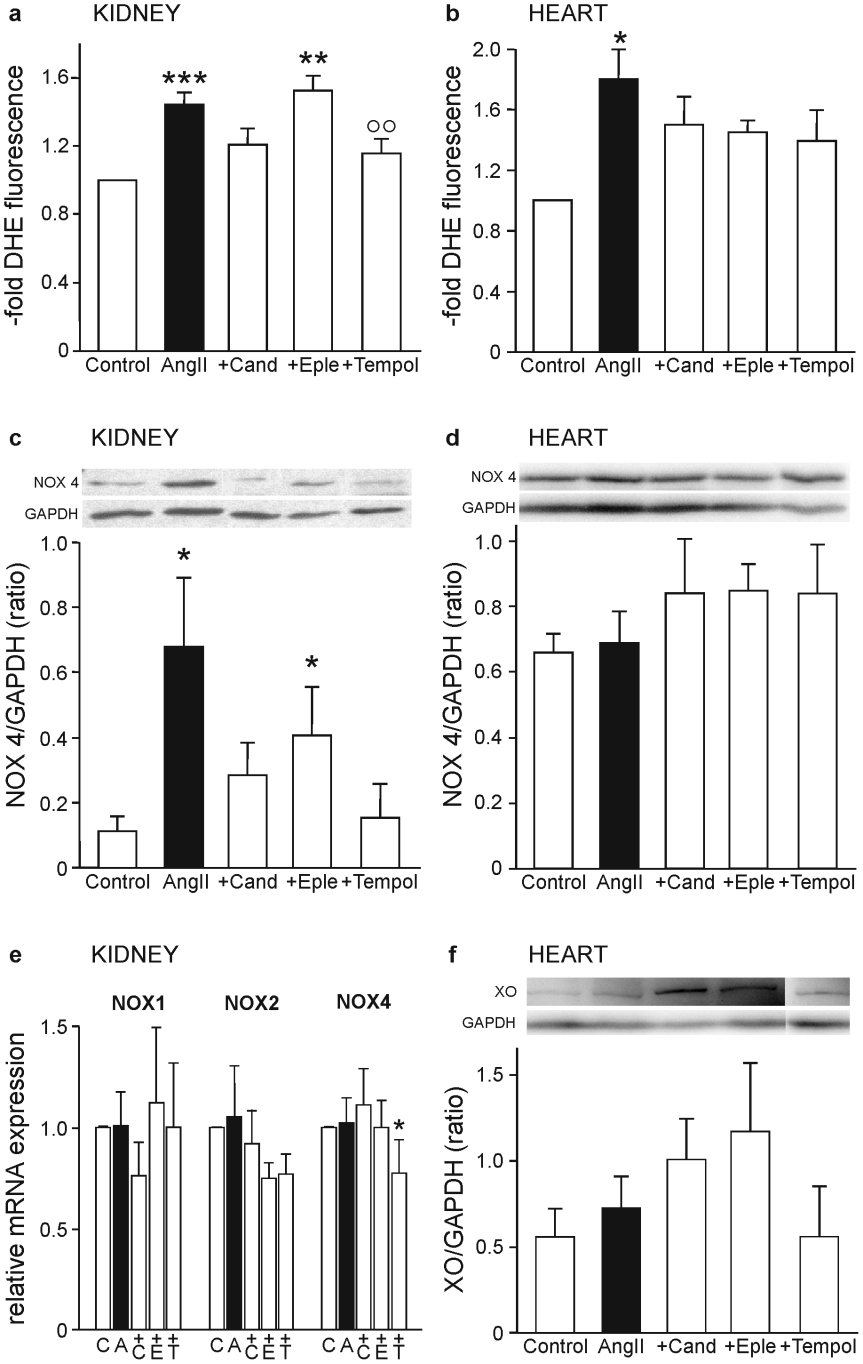
Induction of reactive oxygen species in kidney and heart. ROS formation quantified after staining kidney **(a)** and heart **(b)** cryosections with the ROS-sensitive fluorescent dye dihydroethidium. Quantification was done with the free software Cell Profiler [15]. Data are shown as mean ± SEM after normalization to control values. Western blot-analysis of the amount of one subunit of the ROS-generating enzyme NADPH-oxidase 4 (NOX4) in protein extracts of kidney **(c)** and heart **(d)**, related to the house-keeping protein glyceraldehyde 3-phosphate dehydrogenase (GAPDH). Shown are representative blots and the quantification of band densities of proteins of all animals. Relative quantification of the transcripts of the NADPH-oxidase subunits 1 (NOX1), 2 (NOX2) and 4 (NOX4) in kidney tissue **(e)**. Western blot-analysis of the amount of xanthine oxidase in protein extracts of the heart **(f)** related to the house-keeping protein glyceraldehyde 3-phosphate dehydrogenase (GAPDH). Shown is a representative blot and the quantification of band densities of proteins of all animals. * p≤0.05, ** p<0.01, *** p<0.001 vs. Control, °° p<0.01 vs. AngII-treatment.

As observed earlier [12], treatment with AngII causes a significant increase of oxidative stress in kidney and heart of AngII-infused mice (Fig. 3a and b). In the kidney, treatment with candesartan and tempol decreased ROS formation, the latter significantly, but eplerenone had no beneficial impact on this organ at all. In the heart, on the other hand, all interventions slightly lowered the amount of ROS. As a possible source of ROS, the expression of the ROS-producing subunit of the NADPH-oxidase isoform 4, Nox4, was analysed. In the kidney, AngII caused a significantly raised expression of this subunit, while no change could be observed in the heart (Fig. 3c and d). Quantifiying the amount of renal mRNA of the three NADPH isoforms Nox1, Nox2 and Nox4, no significant increase of any isoform could be detected (Fig. 3e). Only tempol decreased Nox4 mRNA significantly compared to the control group. As an important oxidative enzyme in the heart, the abundance of xanthine oxidase was analysed, which tended to be higher in all groups compared to the control, except in the tempol group (Fig. 3f).

### Induction of transcription factors

As we could detect oxidative stress induced by AngII, the effect of AngII on the expression of the transcription factor regulating the cellular antioxidative defense, Nrf2 (nuclear factor erythroid 2-related factor 2), and of the proinflammatory transcription factor NF-κB (nuclear factor “kappa-light-chain-enhancer” of activated B-cells) was studied.

**Figure 4:**
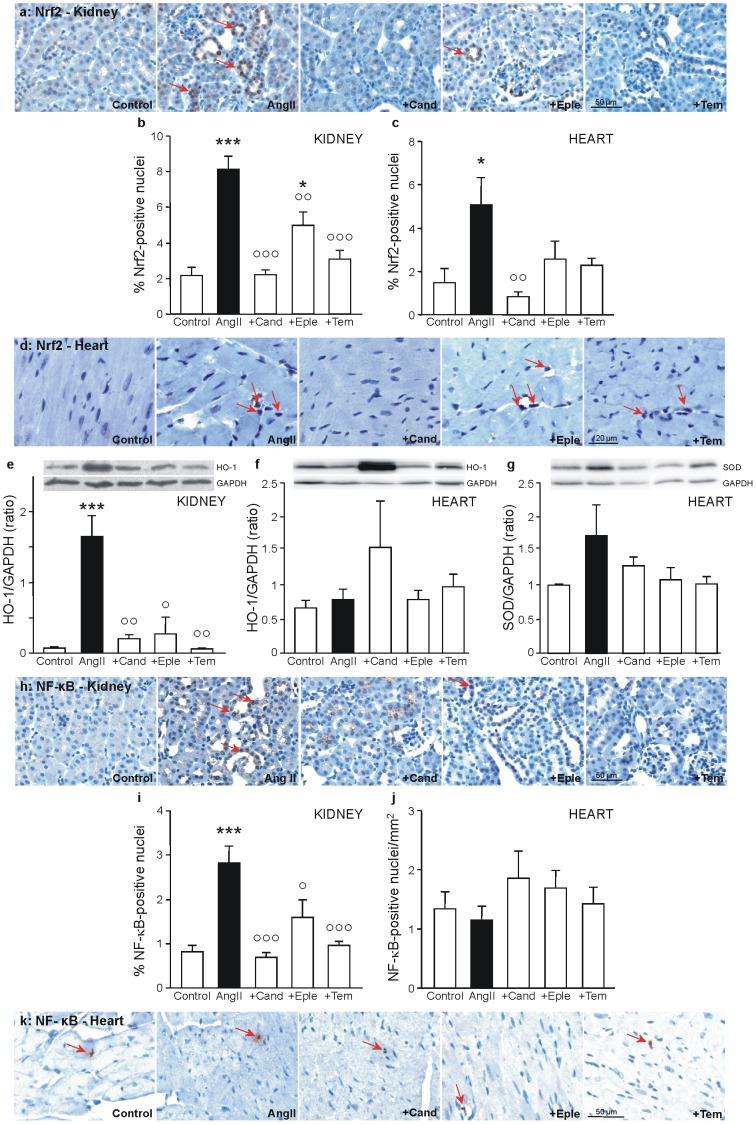
Induction of transcription factors and target proteins. Shown are representative pictures of paraffin-embedded kidney cortex and heart sections of control animals and animals treated with angiotensin II (AngII) with or without co-treatment with candesartan (+Cand), eplerenone (+Eple), or tempol (+Tem). Some examples of positive-stained cells are marked with red arrows. Representative pictures of the immunohistochemical detection of (**a**) Nrf2-positive cells on kidney sections. Quantification of cells positively stained for Nrf2 with the cell image analysis CellProfiler [15] on kidney (**b**) and heart (**c**) tissue in 7 visual fields. Representative pictures of the immunohistochemical detection of Nrf2-positive cells on heart sections (**d**). Western blot-analysis of the amount of the Nrf2-target protein heme oxygenase-1 (HO-1) in protein extracts of kidney (**e**) and heart (**f**), and the Nrf2-target protein superoxide dismutase (SOD) in protein extracts of heart (**g**), related to the house-keeping protein glyceraldehyde 3-phosphate dehydrogenase (GAPDH). Shown are representative blots and quantification of band densities of proteins of all animals. Representative pictures of the immunohistochemical detection of (**h**) NF-κB-positive cells on kidney sections. Quantification of cells positively stained for NF-κB with the cell image analysis CellProfiler [15] on kidney tissue in 7 visual fields (**i**). Quantification of cells positively stained for NF-κB per mm^2^ of heart tissue (**j**). Representative pictures of the immunohistochemical detection of (**k**) NF-κB-positive cells on heart sections. * p≤0.05, ** p<0.01,*** p<0.001 vs. Control, ° p≤0.05, °° p<0.01, °°° p<0.001 vs. AngII-treatment.

Nrf2 is activated by oxidative and electrophilic stress resulting in the translocation of Nrf2 to the cell nucleus and the expression of antioxidative proteins. As can be seen in Fig. 4a-d, AngII caused a significant increase of Nrf2-positive nuclei in kidney and heart, evidence for the presence of oxidative stress in these organs. Candesartan was able to completely prevent Nrf2-activation in kidney as well as in the heart, tempol and eplerenone also prevented the activation, reaching significance only in the kidney. Again eplerenone was the least efficient intervention. To assess Nrf2-activation beyond mere translocation, the expression of a typical target gene of Nrf2, the antioxidative heme oxygenase-1 (HO-1) was analysed by western blotting in kidney and heart tissue (Fig. 4e and f). HO-1 showed a significant rise in expression in the kidneys of AngII-treated animals, which was reduced by all interventions. In the heart no increase of HO-1 could be observed, except of a non-significant rise in the candesartan-treated group. Another target gene of Nrf2, superoxide dismutase, showed a tendency to be higher in the hearts of the AngII-treated animals (Fig. 4g).

Oxidative stress is one of the activators of NF-κB, leading to the release of NF-κB from cytosolic inhibitors, to its translocation to the nucleus and subsequently to the expression of NF-κB-target genes. AngII caused a significant enhancement of NF-κB-positive nuclei in kidney, which was ameliorated by all interventions in the kidney (Fig. 4h and i). In the heart no effect of AngII on the activation of NF-κB could be detected (Fig. 4j and k).

### DNA-damage in kidney and heart

**Figure 5:**
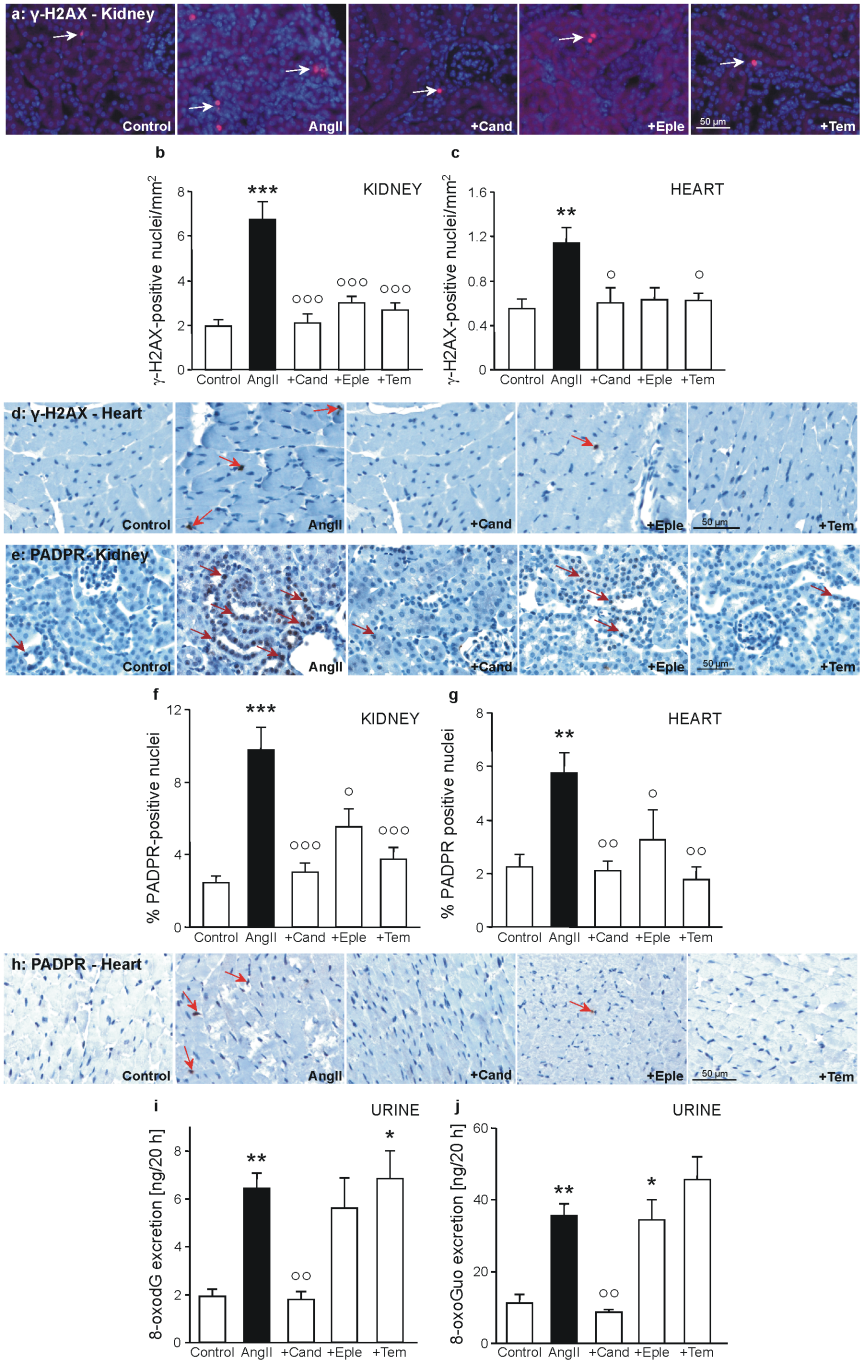
Markers of DNA damage and DNA repair in kidney, heart and urine. Shown are representative pictures of paraffin-embedded kidney cortex and heart sections of animals of the Control group, the angiotensin II group (AngII), the AngII/candesartan group, the AngII/eplerenone group, and the AngII/tempol group. Some examples of positive-stained cells are marked with white (**a**) or red (**a, e, h**) arrows. Representative pictures of the immunofluorescent detection of (**a**) γ-H2AX-positive cells on kidney sections. Quantification of cells positively stained for γ-H2AX per mm^2^ of kidney (**b**) and heart (**c**) tissue. Representative pictures of the immunohistochemical detection of γ-H2AX -positive cells on heart sections (**d**). Representative pictures of the immunofluorescent detection of (**e**) poly(ADP-ribose) (PADPR)-positive cells on kidney sections. Quantification of cells positively stained for PADPR with the cell image analysis CellProfiler [15] on kidney tissue (**f**) and heart tissue (**g**) in 7 visual fields. Representative pictures of the immunohistochemical detection of PADPR-positive cells on heart sections (**h**). As a marker of DNA oxidation, excreted 7,8-dihydro-8-oxo-2′-deoxyguanosine (8-oxodG) was measured in urine by mass spectrometry (**i**). As a marker of RNA oxidation, excreted 7,8-dihydro-8-oxo-guanosine (8-oxoGuo) was measured in urine by mass spectrometry (**j**). * p≤0.05, ** p<0.01,*** p<0.001 vs. Control, ° p≤0.05, °° p<0.01, °°° p<0.001 vs. AngII-treatment.

Although the induction of Nrf2 implies that the cells might be protected against oxidative damage due to an increased antioxidative potential, DNA damage and elevated DNA repair activity was detected in kidney and heart. DNA double strand breaks were immunostained with an antibody against the DNA double strand break marker γ-H2AX. As can be seen in Fig. 5a-d, AngII-treatment resulted in a significantly increased amount of DNA double strand breaks in kidney and heart. All interventions were able to reduce this particular DNA damage to control level, with all of them reaching significance, except eplerenone in the heart.

To analyze the impact of AngII on DNA repair, the activity of poly(ADP-ribose) (PADPR)-polymerases (PARP) was evaluated. PARP, known as an important enzyme in cellular apoptosis, also has a fundamental role in DNA repair. It binds to DNA single strand breaks and synthesizes PADPR chains as a recruting signal for other DNA repair enzymes. In heart and kidney of AngII-treated mice, a significant induction of PADPR-chain synthesis could be found, a further sign of the occurrence of DNA strand breaks (Fig. 5e-h). All interventions did reduce the amount of PADPR chains significantly.

Quantification of oxidized nucleosides and ribonucleosides in urine as a marker of oxidative stress to DNA and RNA bases revealed a significantly increased oxidation in AngII-treated animals, which was prevented by candesartan, but not by eplerenone or tempol (Fig. 5i and j).

## Discussion

Recently we could show that AngII-treatment increases oxidative stress and structural DNA damage dose-dependently *in vivo* in kidney and heart of mice [12]. *In vitro*, we detected important roles of AT1R and Nox4 in the generation of AngII-induced DNA damage [14]. The present study analyzed these aspects *in vivo*, including the impact of increased blood pressure on the studied endpoints. Additionally, the possible participation of AngII-stimulated increased aldosterone levels, a substance also shown to be genotoxic *in vitro* as well as *in vivo* [11], [13], was examined.

### AngII causes oxidative DNA damage and induces stress-related transcription factors

The changes of clinical parameters induced by AngII, like the increase of blood pressure and the loss of kidney function, as well as the histological alterations, were expected from the literature (for example [19]), and from experience gained in earlier experiments [12]. The increase of oxidative stress in the kidney, already seen in a dose-effect experiment [12], could be confirmed with the chosen concentration, which also led to a significant higher production of ROS in the heart. As a source of ROS in AngII-treated cells *in vitro*, we could identify the NADPH oxidase, and in particular the isoform Nox4 [14]. *In vivo*, in the kidney, Nox4 protein also was expressed at higher levels in AngII-infused animals compared to the control, but not in the heart. mRNA levels of Nox4 in the kidney were not markedly increased. The reason for this is not known, but might be due to a temporal sequence of upregulation and downregulation of the enzyme, which is controlled via its mRNA levels [20]. In other hypertension models, higher levels of Nox4 protein (spontaneously hypertensive rat [21],), or Nox4 mRNA (AngII-infusion, mouse [22]), or both (ren2-rat [23]) were found in the kidney. Since in models of kidney disease, Nox4 was shown to rather have a protecting than a damaging role [24], an alternative explanation to a participation of Nox4 in the production of oxidative stress might be, that Nox4 is upregulated in AngII-treated animals as a consequence of their kidney injury or their oxidative damage and not as the source. In the heart as well, the current state of research implies a protective role of Nox4 [25]. In the present study this protective role was probably not accomplished, since no increased protein level could be found. Another oxidative enzyme important in the heart, xanthine oxidase [26] neither did show a significant upregulation by AngII. While increased levels of xanthine oxidase were found in heart failure [27], in the salt-sensitive hypertensive rat too, no significantly higher activity of this enzyme compared to prehypertensive animals was found [28]. In summary, in this study the source of the oxidative stress caused by AngII in the heart could not be identified ultimately.

The detected increase of nuclei positive for the transcription factor Nrf2 in kidneys and hearts of AngII-treated animals underlines the treatment-mediated formation of oxidative stress in these organs, since Nrf2 is activated by ROS and coordinates the antioxidant defense [29]. While Nrf2 activation was not studied yet in AngII-induced hypertension, data from other hypertension models are conflicting. In mineralocorticoid-dependent hypertension Nrf2 mRNA and protein, as well as the target protein heme oxygenase-1 were upregulated, as seen in our mice [30], [31]. In the spontaneously hypertensive rat, the Nrf2 response was impaired [32], just as in models of kidney disease [33], [34]. A possible explanation for the discrepancies between the results of the different hypertension models might be the treatment time, since our study and the mineralocorticoid study were conducted for 4 and 5 weeks, respectively, while the studies in the spontaneously hypertensive rat took 12 and 14 weeks. Maybe Nrf2 was first upregulated, and during chronic oxidative stress, downregulated again. *In vitro*, on the other hand, a suppression of Nrf2 signaling by AngII was already found after 24 hours [35]. In the present study, just as in rats with aldosterone-mediated hypertension [31], the Nrf2-activation was not sufficient to protect the organs from oxidative DNA damage induced by the AngII-dependent hypertension. Concerning the activation of target genes of Nrf2, we found different responses in kidney and heart. In kidney the expression of HO-1 was induced, as seen in the AngII-induced hypertensive rat [36]. Although in a similar model of AngII-infusion, HO-1 upregulation was also observed in the heart [37], here this was not the case. Instead, we saw an upregulation of superoxide dismutase, another Nrf2 target gene.

In the kidneys of our mice, an activation of the pro-inflammatory transcription factor NF-κB could be observed, which was shown already for the model of AngII-infused mice [38]. Unlike other studies examining NF-κB- and Nrf2-activation simultaneously, showing an increase of NF-κB-activity accompanied by a decrease of Nrf2-activity [39], [40], we detected an increase in both activities. It is known that depending on the amount of ROS or DNA damage produced in the cell, an active NF-κB contributes to the survival of the stressed cell [41], just as the activated Nrf2. Thus, an activation of both signaling pathways in kidney cells with DNA damage might permit the survival of a potentially mutated cell. In the heart of AngII-infused mice different results concerning changes in the activation of NF-κB were published: either no direct induction was observed as in our study (Xu et al. 2011 found a phosphorylation of the inhibitor [42]), or, when using the more sensitive EMSA, an increased DNA binding of NF-κB initiated by AngII could be seen. But those were studies with shorter treatment times of 30 minutes and 7 days, respectively [43], [44].

AngII-treatment caused DNA double strand breaks in kidney and heart. This marker of DNA damage was more clearly increased than the oxidative stress, which seems to be conflicting, but can easily be explained by the nature of the markers analyzed. Staining for ROS with the fluorophore dihydroethidium only reflects instantaneous production of ROS on tissue slices. Detection of double strand breaks is based on a posttranscriptional modification of histone proteins in the vicinity of DNA lesions, accumulating over time. Therefore, the impact of AngII-treatment seems to be higher when looking at double strand breaks. The results of DNA repair activity of PARP, the enzyme involved in the repair of DNA strand breaks, in kidney and heart are consistent with the occurrence of DNA double strand breaks. Further, an increase of excretion of oxidized nucleosides (8-oxodG) and ribonucleosides (8-oxoGuo) was measured in urine from angiotensin II-treated animals. 8-oxodG and 8-oxoGuo are guanine nucleoside species produced upon oxidation of guanine nucleotides in DNA and RNA, respectively, and excreted unchanged into the urine [45]. Measuring the nucleosides guarantees that species originating only from the body's cells and not from the diet are quantified [46]. Since these markers are comparatively new, not many studies are published yet. 8-oxodG and 8-oxoGuo are currently being validated as biomarkers for prediction and prognosis in diabetes mellitus [45]. In patients with cardiovascular disease, a higher abundance of urinary 8-oxodG levels was observed [47]. The results obtained in hypertensive individuals are contradictory at the moment: they range from no correlation between 8-oxodG excretion and blood pressure levels [48], [49], to a positive correlation [50] and even a negative correlation [51]. This is the first report showing an increase of urinary 8-oxodG and 8-oxoGuo in a mouse model of hypertension. In rat models the treatment leading to hypertension also increased urinary 8-oxodG [52]–[54].

### Impact of receptor-antagonism

All the above mentioned effects of AngII were prevented by blocking the AT1R with candesartan. For the first time this is reported for the induction of DNA damage in kidney and heart, the induction of DNA repair, the measurement of oxidized bases in urine and the activation of Nrf2. In the cases of endpoints measured in the heart which were not significantly increased by AngII, of course no effect of candesartan could be seen.

Blocking the mineralocorticoid receptor could not prevent or reverse AngII-mediated effects as efficiently as blocking the AT1R. For a start, the administration of eplerenone could not reduce the AngII-induced high blood pressure, but this was to be expected from former studies in rats and mice [55], [56]. Further, the decreased kidney function was not ameliorated by eplerenone, which contradicts a study in the stroke-prone spontaneously hypertensive rat, where eplerenone was able to prevent proteinuria and renal lesions [57]. In humans, treatment of hypertension with a mineralocorticoid receptor antagonist additionally to an angiotensin I-converting enzyme blocker or an AT1R-blocker, also resulted in reduction of albuminuria/proteinuria [58]. In the uninephrectomized rat treated with AngII on the other hand, MR antagonism also could not normalize albumin loss [59]. Aldosterone is known to cause podocyte injury, resulting in damaged glomeruli, which can be prevented by eplerenone [60]. An explanation why in our study albuminuria was not reduced completely by eplerenone might be that AngII was shown to also have adverse effects on podocytes [61]. The observation that glomerular damage cannot be reversed completely by antagonizing the MR, is supported by studies in models with mineralocorticoid-mediated adverse effects showing similar results [13], [62]. Tubular damage induced by aldosterone on the other hand, was reduced to control levels by blocking the MR in our mouse model and in other rat hypertension models [13], [59], [63]–[65]. The beneficial effects of eplerenone on vascular damage probably resulted from inhibition of MR in smooth muscle cells, which on top was shown to be upregulated by AngII [66]. Aldosterone binding triggered vascular contraction, inflammation and remodeling [67]. These effects could be prevented by spironolactone, as shown in rabbit arteries, where remodeling in the form of neointimal thickening induced by aldosterone was inhibited [68]. And also the expression of pro-inflammatory proteins could be reduced by blocking the MR [66].

We can show here for the first time that DNA damage in kidneys and hearts of animals infused with AngII could also be reduced quite efficiently by blocking the MR, indicating a role for both AngII and aldosterone in the induction of DNA damage. Beside one study in diabetic mice [69], which to some extent supports the lack of 8-oxodG reduction by eplerenone in our mice, to our knowledge, no further studies measuring the impact of mineralocorticoid-antagonism on 8-oxodG excretion were conducted up to now. And since this is the first study showing the impact of AngII on the excretion of 8-oxoGuo, no knowledge about the excretion of this biomarker exists. The situation of 8-oxodG seems to be different in rats, where eplerenone was shown to reduce urinary 8-oxodG in animals with unilateral ureteral obstruction, a model of renal fibrosis with no increase in blood pressure [70], and also in salt-induced hypertension [71]. Further studies might show a possible difference between mice and rats in the future. An explanation for the failure of eplerenone in reducing urinary excretion of oxidized bases might be that the beneficial effects of MR blockade operate on another level than the prevention of oxidative stress.

This level could be the transcriptional level, where as an example for a potentially adverse acting transcription factor we could show the activation of NF-κB by AngII-induced hypertension and a reduction of this activation by eplerenone. Reducing NF-κB activity can result in decreased kidney injury as seen in rats with mineralocorticoid-dependent hypertension [72] and in lower vascular inflammation as observed in the spontaneously hypertensive rat [73]. In our mice, both, reduced (not normalized!) kidney injury and reduced vascular injury were seen after eplerenone supplementation, which could be explained by inhibition of NF-κB activity.

### Impact of radical scavenging

The ROS-scavenger tempol, like eplerenone, did not lower hypertension, indeed, the diastolic pressure was even increased. In AngII-infused mice, also only a moderate decrease of blood pressure was observed, initiated by tempol [74], whereas other mice studies reported tempol to be completely ineffective in lowering blood pressure [75], [76]. In rat models of hypertension, tempol almost always decreased blood pressure, often down to control levels [77]. But also here exist exceptions, as for example the ren2-rat or the rat with mineralocorticoid-induced hypertension, where tempol treatment was not able to normalize the blood pressure [13], [78]. Renal function measured as creatinine clearance and albumin excretion was not normalized in our study by tempol, as was also the case in the spontaneously hypertensive rat and the AngII-infused rat [79], [80].

In conclusion, this study shows the importance of the two blood pressure-regulating hormones AngII and aldosterone for the development of hypertension-induced oxidative stress and DNA damage. Blocking the AT1R prevented all AngII-mediated oxidative effects, while blocking the MR prevented not all, but crucial damage like the emergence of DNA double strand breaks. AngII caused activation of the transcription factors Nrf2 and NF-κB. Unlike other studies examining both NF-κB- and Nrf2-activation, showing an increase of NF-κB-activity accompanied by a decrease of Nrf2-activity [39], [81], we detected an increase in both activities. It is known that depending on the amount of ROS or DNA damage produced in the cell, an active NF-κB contributes to the survival of the stressed cell [41], just as the activated Nrf2. Thus, an activation of both signaling pathways in kidney cells with DNA damage might permit the survival of a potentially mutated cell, further adding to the hypothesis of a possible carcinogenic effect of AngII.

## Supporting Information

S1 Table **Raw data belonging to** **Tables 3** **and** **4** **and** **Figs. 1** **,** **3** **,** **4** **and** **5** **.** Data in gray: raw data without normalization, data in black: final data included in the manuscript.
(XLS)
